# Supplementary material for: RAM-PGK: Prediction of Lysine Phosphoglycerylation Based on Residue Adjacency Matrix
Source: Genes (Basel). 2020 Dec 20;11(12):1524. doi: 10.3390/genes11121524 (PMC7766696; doi:10.3390/genes11121524)
Supplement: Supplementary file 1 [file genes-11-01524-s001.pdf]

genes

Supplementary File S1

RAM-PGK: Prediction of Lysine Phosphoglycerylation based on Residue Adjacency Matrix

Abel Avitesh Chandra [1,†,*], Alok Sharma [1,2,3,†,*], Abdollah Dehzangi [4,5] and Tatushiko Tsunoda [2,6,7]

1 School of Engineering & Physics, University of the South Pacific, Fiji
2 Laboratory for Medical Science Mathematics, RIKEN Center for Integrative Medical Sciences, Yokohama 230-0045, Japan
3 Institute for Integrated and Intelligent Systems, Griffith University, Brisbane, QLD 4111, Australia
4 Department of Computer Science, Rutgers University, Camden, NJ 08102, USA
5 Center for Computational and Integrative Biology, Rutgers University, Camden, NJ 08102, USA
6 Laboratory for Medical Science Mathematics, Department of Biological Sciences, Graduate School of Science, The University of Tokyo, Tokyo 113-0033, Japan
7 Department of Medical Science Mathematics, Medical Research Institute, Tokyo Medical and Dental University, Tokyo 113-8510, Japan
* Correspondence: abelavit@gmail.com (A.A.C.); alok.sharma@griffith.edu.au (A.S.)
† These authors contributed equally to this work.

Supplement Material. Comparison of the time needed to carry out feature construction and 6-fold cross-validation of each of the methods on Matlab program. The comparison was carried out on a machine with 24 CPUs (Intel(R) Xeon(R) CPU E5 – 2650 v4 @ 2.20GHz) and 125.8 GB memory. PSI-BLAST tool was run using 16 CPUs while the other programs was run on single CPU.

Method	Feature Construction time	6-Fold Cross-Validation time	Total Time (seconds)
CKSAAP_PhoglySite	402.53 sec	83.82 sec	**486.35**
iPGK-PseAAC	6.91 sec	8.69 sec	**15.60**
Bigram-PGK	PSI-BLAST + Matlab Program<br>5078.39 + 15.71 = 5094.10 sec	2.12 sec	**5096.22**
RAM-PGK	48.52 sec	0.49 sec	**49.01**
